# Intra-Uterine and Vaginal Irritation

**Published:** 1887-04

**Authors:** Thos. Lothrop

**Affiliations:** Professor of Obstetrics, Medical Department of Niagara University. Physician to Buffalo Maternity Hospital


VOL. XXVI. APRIL, 1887. No. 9

### Original Communications.

### INTRA-UTERINE AND VAGINAL IRRITATION.*

BY THOS. LOTHROP, M. D.,

Professor of Obstetrics, Medical Department of Niagara University.
Physician to Buffalo Maternity Hospital.

This subject is selected because of its appropriateness in a society devoted to the advancement of the science of obstetrics, and also because of its practical value and interest to the general practitioner. It is too much to expect, however, that any new principle will be enunciated. If in the discussion, which, it is hoped, these few fragmentary thoughts will call forth, even well-established methods of treatment are criticized, and clearer indications for the invasion of the uterine cavity with well-known germicides are made known, the full purpose of the paper will be fulfilled.

Looking back over a period of thirty years, embraced in the professional life of the writer, the revolution in the pathology and therapeutics of pregnancy and the puerperal period seems more like a dream than a reality. If any will recall the professional experience of that period, and review the impotency of medical skill, or, rather, want of skill, in treating what are now termed puerperal diseases, due to septic infection, it will be

* Read before Buffalo Obstetrical Society.

realized how marvelous has been the transformation from the blind gropings of the past into the clearer light of the present.

Semmelweiss, an interne in the Vienna maternity, in 1847, formulated the theory that puerperal fever was the product of absorption of septic matter, and Pasteur later demonstrated that the active principle in the virus was a living organism, one of the microbes of decomposition. The genius of Lister applied the principle of antisepsis to surgery, and Stadfeldt, in the Copenhagen maternity, accomplished a reformation in midwifery by adopting the most careful and systematic antiseptic measures, which reduced, within a brief period, the mortality in lying-in-hospitals from ten to twenty per cent. to less than one per cent.

Still later, the introduction of the mercuric chloride by Tarnier, in 1881, placed in the hands of the accoucher a germicide of rare potency, with which to combat the germs, which these later investigations discovered to be the fons et origo of many, if not all, puerperal diseases.

A consideration also of great weight, the product of earlier teachings, was the sacredness of the uterine cavity and the danger of invading its portals. Besides, the indefiniteness of the indications of treatment in puerperal diseases, on account of the imperfect pathological opinions then prevalent, made the position of the accoucher, in combating the diseases often met with in the lying-in-room, peculiarly unsatisfactory, and his labors negative.

In maternity hospitals, the remark of Fritsch, that "to be laid on the bed of confinement was equal to being delivered to the hangman," has been verified in the alarming mortality which has attended puerperal women when collected together in great numbers.

In this connection, we need also to call attention to the susceptibility of the puerperal woman to septic infection, and this condition is due to hyperinosis of the blood and the irritability of the nervous system, which are developed during pregnancy. The rapid involution in the genitals and adjacent parts, the increased activity of the skin, kidneys, lungs and heart, destined to circulate and eliminate waste material, are important factors in the problem.

It has been estimated that puerperal fever alone has numbered among its victims more of the human race than all the sanguinary wars recorded in history. While this, at first sight, may seem an exaggerated statement, a careful investigation reveals abundant reason for regarding it to be within the limits of possibility, and even probability. If the number of births daily are equal to the aggregate of minutes in the diurnal rotation of the earth, it will be seen at once that septic infection has had the broadest field for its occupation, and the victims who have fallen by the way under the older systems of medical treatment, are among the innumerable hosts, beyond the computation of the human mind.

Let me preface, therefore, the paper herewith presented to the society, by accepting the germ theory in the causation of puerperal disease, and, as a consequence of this pathology, the use of germicides in their treatment.

The puerperal woman presents the most fertile condition for septic infection, more favorable even than any condition resulting from traumatism. It is strange that the medical profession have delayed to recognize this fact until so late a period, and to make use of the measures at hand in combating this pathological condition. This conservatism, in accepting new theories, which run counter to long-established principles, has characterized the profession in other departments of medical science. The present day witnesses a more ready acquiescence in the results of scientific research, and a greater readiness to apply new methods in daily practice.

With these general remarks, which seem pertinent to the subject under consideration, let me now direct your attention to certain local conditions in the puerpera, which are essential to an intelligent understanding of the purposes of irrigation. And, first, the condition of the uterus and, indeed, the entire parturient canal, after the expulsion of the fœtus, claim our special study. If, perchance, it were our privilege to examine a uterus at full term, after it has been relieved of its contents, and the genital tract were exposed, we would wonder, not that septic infection occasionally occurred, but that women are exempt from its danger as often as they are. The endometrium, for a few days after parturition, is covered by a greyish decidua vera, its surface presenting an unhealthy, unclean, diphtheritic look, with shreds of membranes, consisting of small portions of the decidua reflexa—partially detached and often decomposed. The placental disk is raw, irregular, and covered with blood clots, closing the mouth of the uterine sinuses. The uterine cavity is occupied with the bloody lochia, the product of the involution which is going on in the organ, by means of which it returns to its normal size and weight. Passing down to the cervix and os uteri, we find frequently lacerations and abrasions, sufficient time not having elapsed for granulations to set up, and thus to form a safeguard against absorption. If the labor has been tediou s the mucous membrane of the vagina is more or less abraided, the fourchette torn, and, unless great care has been exercised in the passage of the fœtal head, the perineum has suffered injury. Along this tract the lochial discharge passes, running over a broad surface, offering numerous points for its absorption. If, perchance, a poisonous element has entered the genital canal, it finds here the more favorable conditions, both local and general, for its activity and cultivation. The period is too early for the healing process to have started and become so far advanced, that the lacerated surfaces are guarded against absorption, while the endometrium covered with organic matter, in which decomposition has been started, offers a quick avenue for the passage of living organism into the circulation, from which serious and perhaps fatal results may accrue. A normal labor, under the most favorable auspices, may be followed in a few days by symptoms of septic infection, as chills, a severe pyrexia, offensive lochia, etc., and the bright hopes and anticipations, the first outbursts of a safe delivery, are changed to forebodings of danger and alarm. This transformation may occur even after the most careful attention to details on the part of the accoucher.

These unfavorable changes are more frequent and dangerous in cases where the natural progress of gestation has been interrupted and abortion or premature labor takes place. Here the retention of portions of the placenta is more likely to occur, and, besides, the system is not prepared for the change which takes place as a result of the discharge of the fœtus at an earlier period than full time, and, therefore, falls an easy prey to the insidious entrance of the microbe of decomposition. Nature is exceedingly jealous in her oversight of the reproductive organs, and manifests her displeasure at any interference with their office in the economy of nature, by visiting the victim with deserved chastisement. Whether it be septic infection or pelvic inflammation, the source is the same—the introduction of the products of decomposed organic matter.

While septic infection offers the most general and frequent indication for the use of uterine irrigation, there are other conditions following parturition in which it is specially called for. The use of *hot water* in hemorrhage is a recognized procedure in surgery, and the accoucher has been ready to borrow, from a co-ordinate department of medical science, one of the most efficient means for arresting post partum hemorrhage. The fact that hot water is always at hand, and its reliability as a hæmastatic generally known to the profession, calls for its use in cases of uterine inertia or the hemorrhagic diathesis, in which severe flowing follows the expulsion of the fœtus, as the most efficient means for its arrest. The irrigation of the uterus with water of a temperature of 110° to 115°, will be followed often by contraction of the organ, and the arrest of the hemorrhage, and the rescue of the patient from death.

The use of hot water in softening the rigid os and cervix uteri, in labors in which the necessity is urgent for prompt delivery, on account of eclampsia, etc., is a well-recognized and efficient method of treatment. In cases where other means fail, the irrigation of the cervix with water of a temperature of 110°, continued for one or even two hours, until the rigidity is overcome, is attended with such happy results, that it is commended earnestly to your attention. A case is recalled of a patient at full term with uræmic convulsions, in whom dilatation was quickly accomplished by vaginal irrigation, after chloroform, morphia and atropia had failed, and a forceps-delivery of a dead baby resulted. The utility of this measure is beyond question.

It is, however, in septic infection that vaginal and intra-uterine irrigation find their greatest and most frequent use. Here the results from their judicious employment are marvelous. It is not too much to assert that thousands of women have been saved to enjoy the blessings and joys of maternity, for which they had suffered during a long pregnancy and a painful parturition.

The symptoms indicating the use of irrigation are mainly the presence of a chill succeeded by fever, following within twenty-four or forty-eight hours or even later after delivery; and an interesting question here presents itself—what temperature shall determine the accoucher to order the intra-uterine irrigation? If following a chill, the temperature rises to 101° or 102°, and the pulse corresponds in frequency, and the medical attendant is convinced that septicæmia is the cause of the pyrexia, the thorough washing out of the uterine cavity is indicated. It may be inquired here—how frequently is it safe to repeat the irrigation? and in reply, let me say, that the rise of the temperature, after the first irrigation, is an omen that calls for the repetition of the washing. Some doctors are so enthusiastic as to recommend the constant irrigation of the uterine cavity in severe cases. This can be justified only in desperate cases. In the majority of patients, the intra-uterine washing can be repeated every four hours and more often two or three times per day will be ample to control the pyrexia. Often the wire curette will successfully supplement the irrigation, by removing decomposed organic matter, which the water fails to dislodge.

A simple tube of hard gutta percha and a fountain syringe are all that is necessary to carry out the treatment. The Chamberlain tube of glass has long since been discarded in my practice, and an inexpensive hard rubber tube is used, which is thrown away at the completion of each case, in order to be sure that the disease may not be communicated to other patients. Certain precautions are necessary to be observed in the use of the tube. The introduction should be with care and gentleness; and before the water is allowed to pass into the uterine cavity, the air should be carefully expelled from the tube.

It may be asked in this connection—is there danger in the use of this method of treatment? and I answer, that fatal results have in rare cases attended its use. Besides, secondary uterine hemorrhage has followed the irrigation, the thrombus formed at the mouths of the uterine sinuses having become detached. There is also danger of forcing the antiseptic lotion into the circulation, by introducing the distal end of the tube into the mouth of a uterine sinus. The injection of the fluid into the peritoneal cavity through the fallopian tube, has been regarded as a danger. But these accidents, while they should be recognized, should not deter the accoucher from the only method now known, to reach the source of the septic material, and to remove it from the uterus.

In this connection, the germicide to be employed is a matter of importance. Let me give the proportions of some of the more efficient remedies:

1 to 4,000 of Biniodid. Hydrarg. requires 3 21-25 grains of Biniodid., Potass. Iodid. 2½ gr., water 1 quart.

1 to 8,000 of Biniodid. Hydrarg. requires 1 23-25 grains Biniodid., Potass. Iodid. gr. j, water 1 quart.

1 to 15,000 of Biniodid. Hydrarg. requires 1 6-2500 grains Biniodid., Potass. Iodid. gr. ¾, water 1 quart.

1 to 2,000 Bichl. Hydrarg. requires 7.7 grain Bichl. in one quart of water.

The use of the iodoform suppository, following the intra-uterine irrigation, efficiently supplements the treatment. Dr. Garriques recommends a suppository, composed of one hundred grains of iodoform, and finds valuable results to follow its use.

Let me here suggest that a smaller quantity, a scruple for instance, be used at first. The comparative value of the mercuric preparations need not be considered in this connection. The bichloride is more irritating and less expensive. The biniodide is safer, more powerful, but more expensive. We have been accustomed to use the bichloride, and have referred to the use of the biniodides from the experience of others.

The use of carbolic acid as an antiseptic for our instruments is valuable. Boro-glyceride, boracic acid, salicylic acid, potass. permanganate, and other germicides have been brought to the notice of the profession, but the mercuric preparations have been so satisfactory and efficient, that we regard the other germicides as auxiliary agencies to be used in case there should be any idiosyncrasy on the part of the patient, contra-indicating the mercuric salts.

It may be asked in this connection, if there is danger of the constitutional effects of mercury in the free local use of its compounds, as we have advised. The writer has witnessed one case only of ptyalism from the use of the mercuric chloride. In the many cases in which he has used these germicides, no unpleasant results have been experienced, except the one mentioned. It is well to be watchful for the constitutional effects of these powerful agents, but the danger is limited, and should not deter the accoucher from their use.

A simple precaution should be observed in the use of the intra-uterine douche. The cervix and os should be sufficiently dilated to permit the egress of the fluid, and if contraction of the uterine canal has gone so far that the tube cannot be easily introduced, the uterine dilator should precede the tube, so that ease of introduction and free egress may be maintained.

The use of vaginal irrigation in puerperal cases should be referred to in this paper. It was the practice, a few years ago, to wash out the vagina in all cases. The necessity for this procedure is not apparent. Wherever there is observed an offensive lochia, or a feeling of discomfiture from the presence of clots in the vagina, the use of the vaginal douche will be a source of safety and of comfort to the patient; but care should be exercised in their use. Uterine colic has often resulted from the ingress of the fluid into the uterine canal, too much force having been employed in injecting the fluid, or the distal end of tube placed so near the os, that the uterus has received a larger share of the medicated lotion than its needs called for, and it has simply rebelled.

It is quite beyond the purpose of this paper to call attention to the various diseases in which irrigation may be indicated. The current of medical thought, at this time, turns towards the septic origin of nearly all the diseases arising in the puerperal period. If, therefore, sepsis is accepted as the cause of these complications, the use of germicides internally and externally along the genital tract will be indicated in their successful treatment. At least it may with truthfulness be affirmed, that already a great revolution has been accomplished in the pathology and therapeusis of the puerperal period. How far these opinions will be extended into the domain of many pathological conditions, at present not clearly defined in their limits, it will be too much for the writer to anticipate. But this much may be safely stated, that sepsis offers the only basis upon which to found a rational pathology of puerperal diseases, and if so, the use of antiseptics locally and constitutionally offers the surest means for curative results.

